# Effects of Antiarrhythmics on Cardiac Phenotype and Redox Balance in Spontaneously Hypertensive Rats

**DOI:** 10.3390/ijms27146490

**Published:** 2026-07-21

**Authors:** Stefan M. Simović, Ivan M. Srejović, Goran T. Davidović, Maja D. Murić, Slobodanka L. Mitrović, Marko P. Ravić, Marijana M. Andjić, Marina R. Nikolić, Katarina G. Mihajlović, Nevena D. Lazarević, Sergey B. Bolevich, Aleksandra S. Orlova, Marina A. Fokina, Sergey E. Mironov, Vladimir ǈ. Jakovljević, Jovana N. Novaković

**Affiliations:** 1 Department of Internal Medicine, Faculty of Medical Sciences, University of Kragujevac, 34000 Kragujevac, Serbia; medicusbg@yahoo.com; 2 Clinic for Cardiology, Clinical Center Kragujevac, 34000 Kragujevac, Serbia; 3 Department of Physiology, Faculty of Medical Sciences, University of Kragujevac, 34000 Kragujevac, Serbia; majanikolickg90@gmail.com (M.D.M.); marina.rankovic.95@gmail.com (M.R.N.); drvladakgbg@yahoo.com (V.ǈ.J.); 4 Center of Excellence for the Study of Redox Balance in Cardiovascular and Metabolic Disorders, University of Kragujevac, 34000 Kragujevac, Serbia; smitrovic@fmn.kg.ac.rs (S.L.M.); markoravic@hotmail.com (M.P.R.); andjicmarijana10@gmail.com (M.M.A.); katarina.mihajlovic@fmn.kg.ac.rs (K.G.M.); nevenasdraginic@gmail.com (N.D.L.); jovana.novakovic@fmn.kg.ac.rs (J.N.N.); 5 Department of Pharmacology, First Moscow State Medical University I.M. Sechenov, Moscow 119435, Russia; mironov_s_e@staff.sechenov.ru; 6 Department of Pathology, Faculty of Medical Sciences, University of Kragujevac, 34000 Kragujevac, Serbia; 7 Department of Pharmacy, Faculty of Medical Sciences, University of Kragujevac, 34000 Kragujevac, Serbia; 8 Department of Pathophysiology, First Moscow State Medical University I.M. Sechenov, Moscow 119435, Russia; bolevich2011@yandex.ru (S.B.B.); orlovaas@yandex.ru (A.S.O.); fokina_m_a@staff.sechenov.ru (M.A.F.)

**Keywords:** arterial hypertension, antiarrhythmics, dronedarone, amiodarone, dofetilide, cardiac function, oxidative stress, HSP70, SERCA2

## Abstract

Arterial hypertension is a well-known risk factor for cardiac arrhythmias, while certain antihypertensive agents may also create conditions for arrhythmia occurrence. The aim of this study was to evaluate the effects of selected class III antiarrhythmic drugs on cardiac function and structure, as well as on oxidative stress status, in spontaneously hypertensive rats (SHRs). Male SHRs were treated for 4 weeks with dronedarone, amiodarone, or dofetilide. Cardiac function was assessed in vivo by echocardiography and ex vivo using the Langendorff model. Oxidative stress markers, myocardial morphology, and HSP70 and SERCA2 expression were analyzed. All drugs partially improved cardiac function in vivo, with dronedarone exerting the strongest antihypertensive effect. Ex vivo, dofetilide enhanced contractility and dronedarone reduced cardiac performance, whereas amiodarone showed intermediate effects. Oxidative stress was differentially modulated, with dofetilide and amiodarone improving antioxidant defenses, while dronedarone showed heterogeneous effects. Histologically, dofetilide preserved myocardial architecture most effectively, while amiodarone and dronedarone partially attenuated hypertensive remodeling. Treatments decreased HSP70 expression, while increasing SERCA2 expression, particularly in the dofetilide group. Class III antiarrhythmic drugs differentially affect cardiac function, redox balance, calcium handling, and myocardial remodeling in hypertension, which may contribute to their distinct antiarrhythmic and cardioprotective properties.

## 1. Introduction

Arterial hypertension (HTA), the most common chronic non-communicable disease worldwide, is a well-recognized risk factor for numerous cardiovascular conditions, including cardiac arrhythmias [1]. The pathogenesis of arrhythmias in the field of HTA is complex and involves alterations in hemodynamics, neuroendocrine regulation, myocardial structure, electrophysiological properties, and ion currents, often associated with left ventricular hypertrophy (LVH) [2]. Considering the wide spectrum of arrhythmias, including atrial fibrillation (AF), supraventricular tachycardia, ventricular ectopic beats, ventricular tachycardia, ventricular fibrillation, and various bradyarrhythmias, as well as the complex molecular mechanisms underlying their development, such as disturbances in ion channel expression, renin-angiotensin-aldosterone system (RAAS) dysregulation, structural remodeling, oxidative stress, and inflammation, numerous antiarrhythmic drugs (AADs) have been developed and classified into several groups [3,4]. Furthermore, certain antihypertensive drugs may contribute to arrhythmia occurrence, primarily through electrolyte disorders [1].

Amiodarone is one of the most frequently prescribed AADs and is generally classified as a class III AAD, although it also exhibits properties of class I, II, and IV AADs [5]. Specifically, amiodarone acts as a nonselective inhibitor of the voltage-gated potassium (K^+^) channel, while also blocking sodium (Na^+^) (class I effect) and calcium (Ca^2+^) channels (class IV effect) and β-adrenergic receptors (class IIa effect) [6]. In several clinical conditions, including heart failure (HF), structural heart disease, and significant LVH, amiodarone is considered one of the first-line therapeutic options [7]. Dronedarone, similar to amiodarone, is a nonselective ion channel blocker with properties corresponding to class I-IV AADs [8]. It is recommended for the treatment of AF in patients with HF with preserved ejection fraction (HFpEF), ischemic heart disease (IHD), or valvular heart disease [8]. Dofetilide, on the other hand, is a selective blocker of Kv11.1 voltage-gated K^+^ channels. Compared to amiodarone, it is associated with lower incidence of overall systemic side effects but carries an increased risk of ventricular arrhythmia due to QT interval prolongation and reduced repolarization reserve [9,10].

Oxidative stress, defined as an imbalance between the production and elimination of reactive oxygen species (ROS) and reactive nitrogen species (RNS), resulting from increased ROS/RNS generation or/and insufficient antioxidative defense system, represents an important factor in the development and progression of cardiac arrhythmias. Increased ROS/RNS production within vascular structures and the resulting oxidative damage play an important role in the pathophysiology of HTA [11,12]. Hallmarks of HTA, including endothelial dysfunction, subclinical inflammation, mitochondrial dysfunction, and impaired nitric oxide (NO) bioavailability, further promote the development of a pro-oxidative environment. Mitochondria represent one of the major sources of intracellular ROS production due to their excessive oxygen consumption [13]. ROS and RNS can promote a proarrhythmic environment through alterations in intracellular Ca^2+^ handling, including the modification of ryanodine receptors (RyRs), inhibition of sarcoplasmic reticulum Ca^2+^-ATPase (SERCA) activity, and lipid modification [13]. In addition, oxidative stress can disrupt the structure and function of gap junctions, thereby contributing to the initiation and maintenance of arrhythmias [14]. Excessive mitochondrial ROS production and their release into the cytoplasm may further amplify ROS/RNS production through mechanisms such as NO synthase (NOS) uncoupling or activation of xanthine oxidase [15]. HSP70 is a stress-inducible molecular chaperone that plays a central role in protein folding, protein quality control, and cellular remodeling processes. By acting as a sentinel chaperone, HSP70 protects cells against a wide range of proteotoxic insults, preventing protein misfolding and aggregation while maintaining cellular proteostasis [16]. HSP70 mainly exhibits cardioprotective potential and its expression usually increases under conditions of oxidative stress and cardiomyocyte damage. However, chronic HSP70 activity can potentiate changes such as myocardial hypertrophy [17].

This study aimed to evaluate and compare the effects of class III AADs (dronedarone, amiodarone, and dofetilide) on cardiac function and structure, as well as oxidative stress status in spontaneously hypertensive rats (SHRs), integrating in vivo and ex vivo cardiac function, morphological, and systemic biochemical assessments to elucidate potential redox-mediated mechanisms underlying their cardiovascular actions.

## 2. Results

### 2.1. Effects of Antiarrhythmic Treatments on Arterial Blood Pressure in SHRs

SBP and DBP were measured at baseline and after the 4-week treatment protocol. At baseline, all groups exhibited elevated blood pressure, consistent with HTA (SHR group: 173 ± 8/104 ± 4; Dron group: 171 ± 6/102 ± 5; Ami group: 174 ± 7/101 ± 5; Dof group: 174 ± 4/102 ± 6), without significant statistical differences between groups.

**Figure 1:**
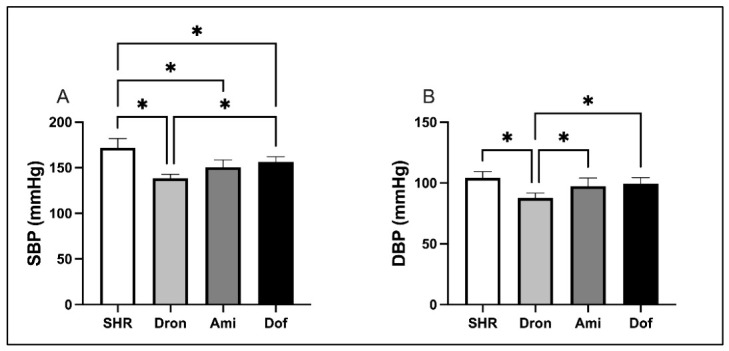
Effects of antiarrhythmic treatments on systolic blood pressure (SBP) and diastolic blood pressure (DBP) in SHRs. (**A**) SBP; (**B**) DBP. Data are presented as the mean ± SD. Statistically significant differences between groups are indicated by *, with significance set at *p* < 0.05.

After the 4-week treatment protocol, the SHR group maintained elevated blood pressure. In contrast, the Ami and Dof groups had significant reductions in SBP (Figure 1A), whereas the Dron group exerted the most pronounced antihypertensive effect, markedly reducing both SBP and DBP compared to the SHR group (Figure 1A,B).

### 2.2. Effects of Antiarrhythmic Treatments on In Vivo Cardiac Function in SHRs

**Figure 2:**
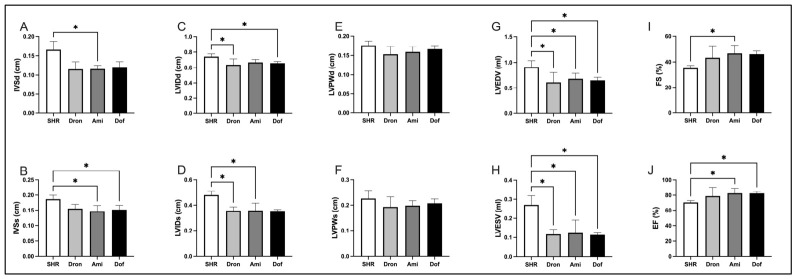
Effects of antiarrhythmic treatments on left ventricular structure and function in SHRs assessed by transthoracic echocardiography. (**A**) Interventricular septal thickness at end-diastole (IVSd); (**B**) interventricular septal thickness at end-systole (IVSs); (**C**) left ventricular internal diameter at end-diastole (LVIDd); (**D**) left ventricular internal diameter at end-systole (LVIDs); (**E**) left ventricular posterior wall thickness at end-diastole (LVPWd); (**F**) left ventricular posterior wall thickness at end-systole (LVPWs); (**G**) left ventricular end-diastolic volume (LVEDV); (**H**) left ventricular end-systolic volume (LVESV). (**I**) Fractional shortening (FS); (**J**) ejection fraction (EF). Data are presented as the mean ± SD. Statistically significant differences between groups are indicated by *, with significance set at *p* < 0.05.

TTE revealed that all three AADs induced comparable alterations in LV structure and function relative to the SHR group, with no significant differences observed among the treated groups (Figure 2). IVSd was significantly reduced in the Ami group compared to the SHR group (Figure 2A), while IVSs were significantly reduced in both the Ami and Dof groups relative to the SHR group (Figure 2B). LVIDd was decreased in the Dron and Dof groups (Figure 2C), whereas LVIDs was decreased in the Dron and Ami groups compared to the SHR group (Figure 2D). LVEDV and LVESV were significantly reduced in all treatment groups relative to the SHR group (Figure 2G,H). FS was significantly increased in the Ami group relative to the SHR group, whereas the other treatment groups exhibited trends toward increased FS that did not reach statistical significance (Figure 2I). EF was elevated in all treatment groups compared to the SHR group, with statistically significant increases observed in the Ami and Dof groups (Figure 2J).

### 2.3. Effects of Antiarrhythmic Treatments on Ex Vivo Cardiac Function in SHRs

**Figure 3:**
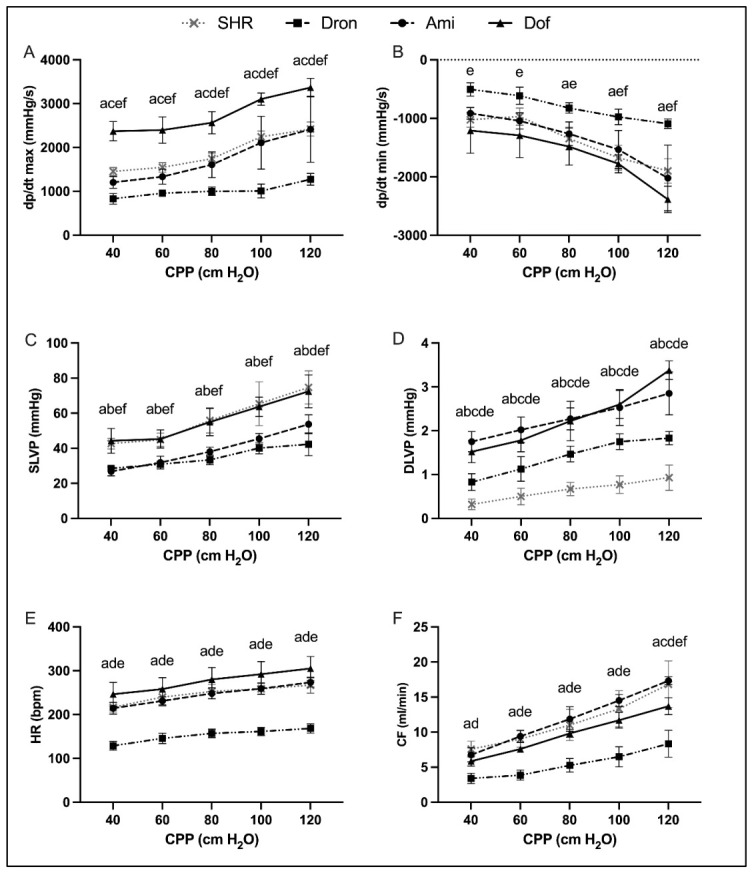
Effects of antiarrhythmic treatments on ex vivo cardiodynamics parameters in SHRs assessed by the Langendorff technique. (**A**) Maximum rate of pressure development in the left ventricle (dp/dt_max); (**B**) minimum rate of pressure development in the left ventricle (dp/dt_min); (**C**) systolic left ventricular pressure (SLVP); (**D**) diastolic left ventricular pressure (DLVP); (**E**) heart rate (HR); (**F**) coronary flow (CF). Data are presented as the mean ± SD. Statistical significance was set at *p* < 0.05. Letters indicate significant differences between groups: ^a^ SHR vs. Dron, ^b^ SHR vs. Ami, ^c^ SHR vs. Dof, ^d^ Dron vs. Ami, ^e^ Dron vs. Dof, and ^f^ Ami vs. Dof.

Ex vivo analysis of cardiac function using the Langendorff technique revealed significant differences in the cardiodynamic parameters between experimental groups across increasing CPP values (40–120 cmH_2_O) (Figure 3).

Compared to the SHR group, the Dof group had significantly increased dp/dt max at all CPP levels, whereas the Dron group had significantly decreased dp/dt max. The Ami group exerted values comparable to the SHR group, without consistent significant deviations. Among the treated groups, the Dof group exhibited the highest dp/dt max values, whereas the Dron group had the lowest values. TheAmi group displayed intermediate values, significantly higher than the Dron group at higher CPP levels (80–120 cmH_2_O) but lower than the Dof group at all CPPs (Figure 3A). For dp/dt min, the Dof group had a more pronounced decrease (more negative values) compared to the SHR group at higher CPPs (80–120 cmH_2_O), while the other groups showed less consistent differences. The Dof group differed significantly from the Dron group at all CPP levels and from the Ami group at higher CPPs (100–120 cmH_2_O) (Figure 3B). The SHR and Dof groups exhibited similar SLVP values throughout the experiment, whereas both the Dron group and Ami group had significantly reduced SLVP across all CPP levels. Among the treated groups, the Dof group showed higher SLVP compared to both the Dron and Ami groups, while at the highest CPP, the Dron group exhibited the lowest values among all groups (Figure 3C). DLVP was significantly increased in all treated groups compared to the SHR group, with the most pronounced effect observed in the Ami and Dof groups. Among the treated groups, both the Ami and Dof groups showed significantly higher DLVP levels than the Dron group (Figure 3D). HR was significantly reduced in the Dron group compared to all other groups at all CPP levels (Figure 3E). The Dron group significantly decreased CF across all CPP levels compared to all other groups, while the Ami group significantly reduced CF only at the highest CPP (Figure 3F).

### 2.4. Effects of Antiarrhythmic Treatments on Coronary Oxidative Status

**Figure 4:**
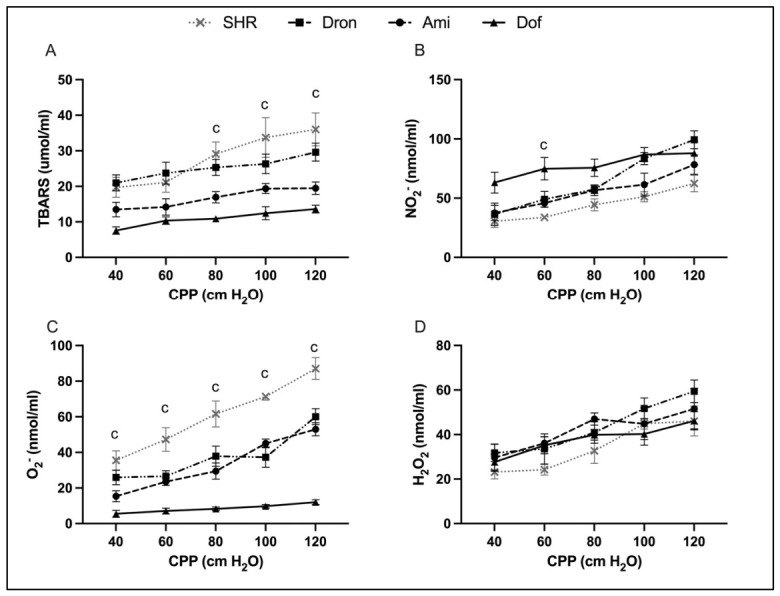
Effects of antiarrhythmic treatments on oxidative stress parameters in coronary venous effluent (CVE) of SHRs. (**A**) Index of lipid peroxidation (measured as TBARS); (**B**) nitric oxide levels expressed as nitrites (NO_2_^−^); (**C**) superoxide anion radical (O_2_^−^); (**D**) hydrogen peroxide (H_2_O_2_). Data are presented as the mean ± SD. Statistical significance was set at *p* < 0.05. Letters indicate significant differences between groups: ^c^ SHR vs. Dof.

Analysis of oxidative stress parameters in CVE revealed significant differences between experimental groups across increasing CPP values (40–120 cmH_2_O) (Figure 4).

The index of lipid peroxidation, measured as TBARS, was significantly reduced in the Dof group compared to the SHR group at higher CPP values (80–120 cmH_2_O). In contrast, the Dron and Ami groups did not exhibit consistent differences relative to the SHR group. Among the treated groups, the Dof group demonstrated the lowest TBARS values at higher CPPs (Figure 4A). NO_2_^−^ values were significantly increased in the Dof group compared to the SHR group only at CPP 60 cmH_2_O, while no significant differences were observed at the remaining CPP levels (Figure 4B). Compared to the SHR group, the Dof group had significantly reduced O_2_^−^ levels at all CPP values (40–120 cmH_2_O), whereas the Dron and Ami groups showed values comparable to the SHR group without consistent significant differences (Figure 4C). H_2_O_2_ levels were comparable among all experimental groups across the entire CPP range, with no consistent significant differences detected (Figure 4D).

### 2.5. Effects of Antiarrhythmic Treatments on Systemic Oxidative Status

**Figure 5:**
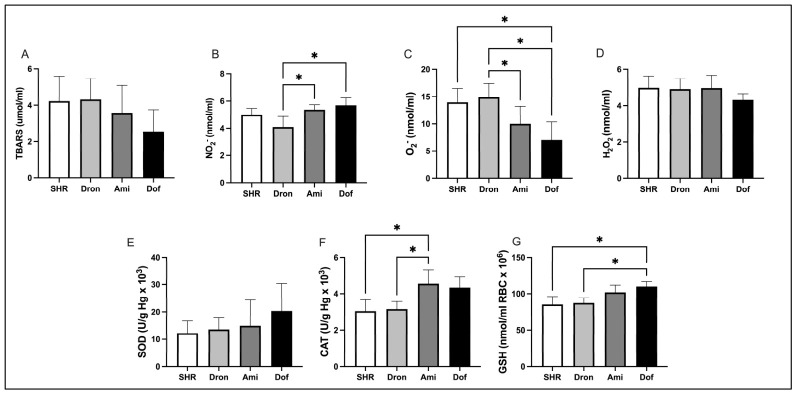
Effects of antiarrhythmic treatments on oxidative stress parameters in the systemic circulation of SHRs. (**A**) Index of lipid peroxidation (measured as TBARS); (**B**) nitrite (NO_2_^−^); (**C**) superoxide anion radical (O_2_^−^); (**D**) hydrogen peroxide (H_2_O_2_), (**E**) superoxide dismutase (SOD); (**F**) catalase (CAT); (**G**) reduced glutathione (GSH). Data are presented as the mean ± SD. Statistically significant differences between groups are indicated by *, with significance set at *p* < 0.05.

The 4-week treatment with AADs resulted in distinct alterations in systemic oxidative stress markers (Figure 5). TBARS levels did not differ significantly among the groups. However, the Dof group exhibited a trend toward lower TBARS levels compared with all other groups (Figure 5A). NO_2_^−^ concentrations were significantly decreased in the Dron group compared to both the Ami and Dof groups (Figure 5B). O_2_^−^ levels were significantly reduced in the Dof group compared to the SHR and Dron groups, and they were also lower in the Ami group relative to the Dron group (Figure 5C). H_2_O_2_ levels were similar and without statistically significant difference between groups (Figure 5D).

Regarding antioxidant defenses, CAT activity was significantly higher in the Ami group compared to the SHR and Dron groups (Figure 5E). In addition, GSH levels were elevated in the Dof group relative to the SHR and Dron groups (Figure 5G).

### 2.6. Effects of Antiarrhythmic Treatments on Histopathological Characteristics of Cardiac Tissue in SHRs

**Figure 6:**
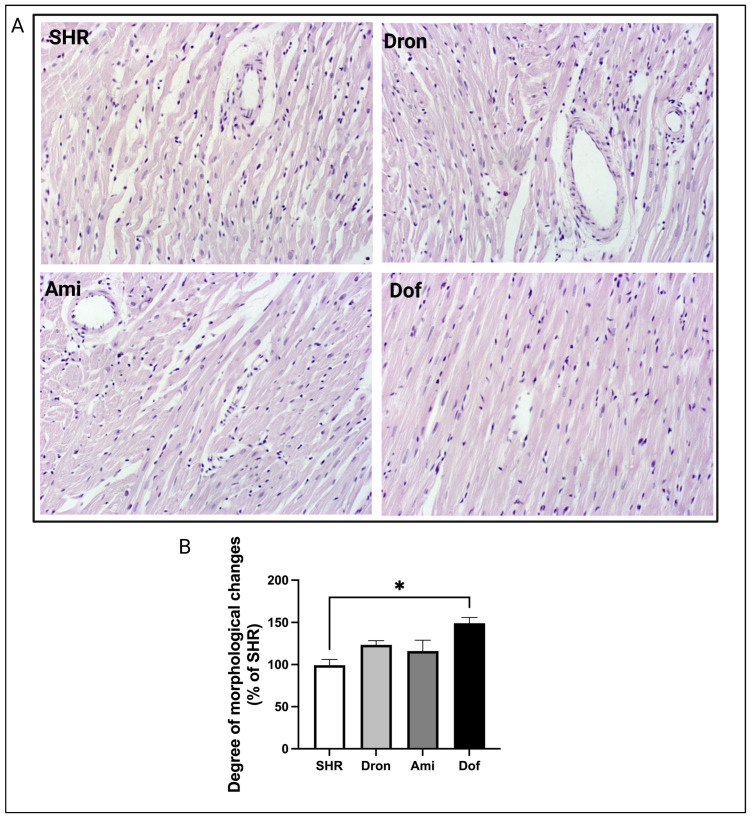
Effects of antiarrhythmic treatments on cardiac histomorphology in SHRs. (**A**) Representative HE-stained myocardial tissue sections (200× magnification). (**B**) The degree of morphological changes (% of SHR). Data are presented as the mean ± SD. Statistically significant differences between groups are indicated by *, with significance set at *p* < 0.05. Created in BioRender: Srejovic, I. (2026) https://BioRender.com/brgun8a, accessed on 19 June 2026.

Histomorphological analysis of hematoxylin and eosin (HE)-stained myocardial sections revealed the most pronounced structural alterations in the SHR group (Figure 6). Myocardial fibers were focally disorganized, with the presence of hypertrophic and attenuated cardiomyocytes, interstitial widening, mild connective tissue expansion, and occasional mononuclear cells. Focal cardiomyocyte necrosis with cytoplasmic hypereosinophilia, nuclear pleomorphism, vesicular nuclei, and occasional perinuclear vacuolization were also observed. In addition, intramyocardial vessels exhibited wall thickening.

The Ami and Dron groups demonstrated similar but less pronounced histopathological alterations, with reduced myocardial disorganization, necrosis, and interstitial changes compared to the SHR group. The Dof group exhibited the mildest histological abnormalities, characterized by relatively preserved myocardial architecture, minimal fibrosis and necrosis, and reduced attenuation of myocardial fibers.

### 2.7. Effects of Antiarrhythmic Treatments on Myocardial HSP70 Expression in SHRs

**Figure 7:**
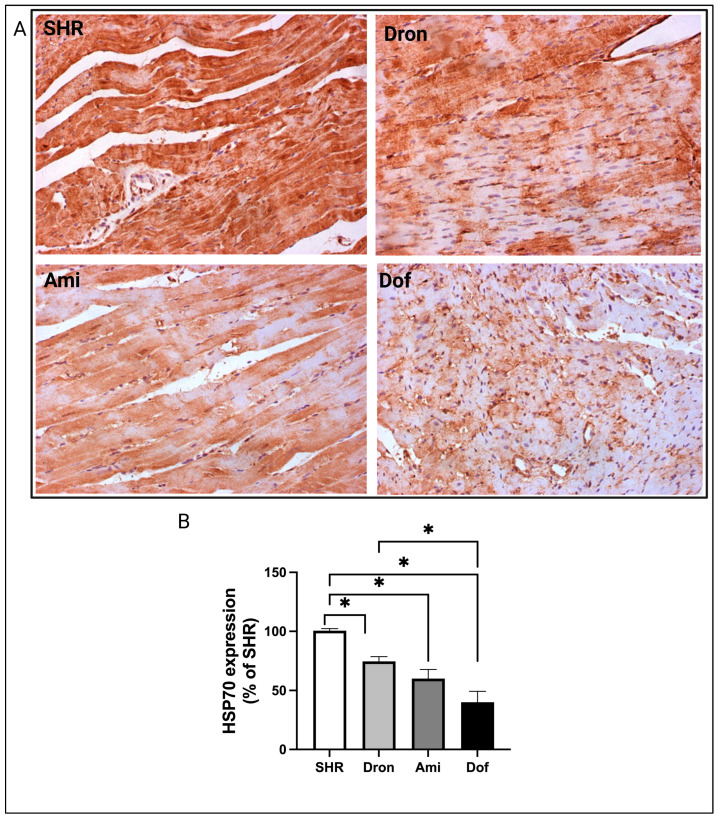
Effects of antiarrhythmic treatments on myocardial HSP70 expression in SHRs. (**A**) Representative ICH-stained myocardial tissue sections (200× magnification). (**B**) HSP70 expression (% of SHR). Statistically significant differences between groups are indicated by *, with significance set at *p* < 0.05. Created in BioRender: Srejovic, I. (2026) https://BioRender.com/vt7qtlv, accessed on 19 June 2026.

Oxidative stress-related changes in myocardial tissue were evaluated by immunohistochemical analysis of HSP70 expression (Figure 7). The highest percentage of cardiomyocytes exhibiting strong HSP70 immunoreactivity was observed in the untreated SHR group, while all treatment groups exhibited significantly lower HSP70 immunoreactivity compared to the SHR group. The Dof group demonstrated the weakest staining intensity and the lowest number of HSP70-positive cardiomyocytes, which was significantly lower than in the Dron group. The Ami and Dron groups showed a comparable pattern of HSP70 expression, although HSP70 immunoreactivity was slightly lower in the Ami group.

### 2.8. Effects of Antiarrhythmic Treatments on Myocardial SERCA2 Expression in SHRs

**Figure 8:**
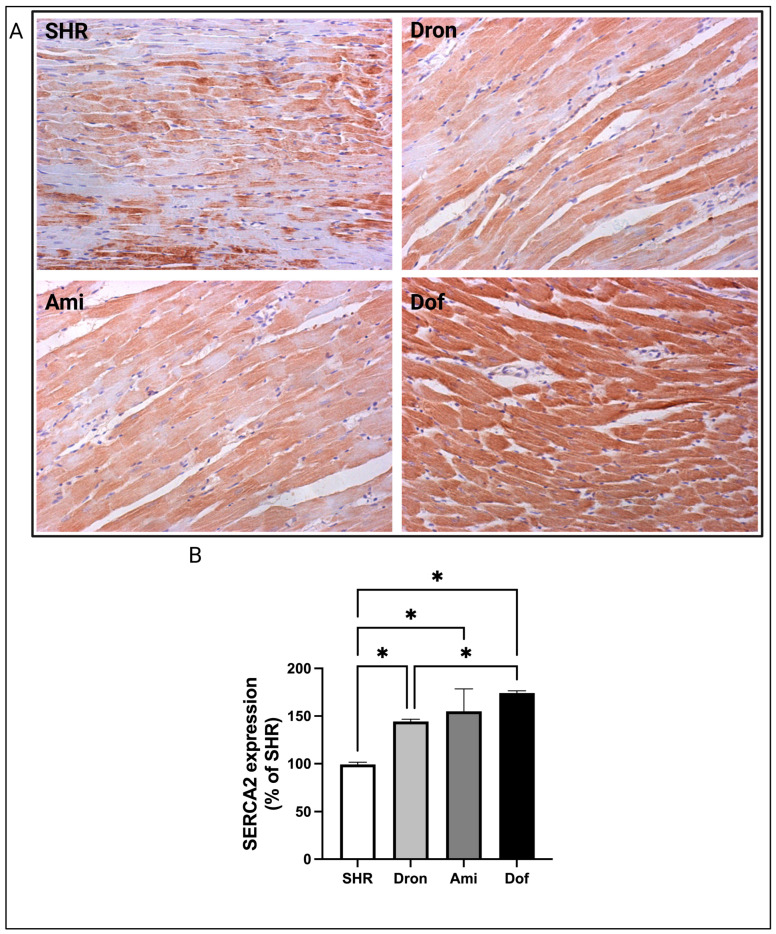
Effects of antiarrhythmic treatments on myocardial SERCA2 expression in SHRs. (**A**) Representative ICH-stained myocardial tissue sections (200× magnification). (**B**) SERCA2 expression (% of SHR). Statistically significant differences between groups are indicated by *, with significance set at *p* < 0.05. Created in BioRender: Srejovic, I. (2026) https://BioRender.com/4xgky6s, accessed on 20 June 2026.

Myocardial contractile function was assessed by immunohistochemical analysis of SERCA2 protein expression in the cytoplasm of cardiomyocytes (Figure 8). The untreated SHR group exhibited the lowest SERCA2 expression levels and the weakest staining intensity in comparison to all treated groups. The percentage of SERCA2-positive cardiomyocytes and the intensity of immunoreactivity were highest in the Dof group, which was significantly different than in the Dron group. Although SERCA2 expression was relatively similar between the Ami and Dron groups, slightly higher immunoreactivity was observed in the Ami group.

## 3. Discussion

The present study provides a comprehensive comparative analysis of three class III AADs—dronedarone, amiodarone, and dofetilide—with a particular focus on their effects on cardiac function, morphology, and redox homeostasis in SHR. By combining in vivo hemodynamic measurements, ex vivo Langendorff assessments, analyses of systemic and coronary oxidative stress markers, and evaluation of structural myocardial changes and immunohistochemical findings, we identified distinct drug-specific profiles that differentially affect myocardial performance and redox regulation.

In vivo, all three agents improved global cardiac performance in SHRs, although with different effects on systemic hemodynamics. Dronedarone exerted the most pronounced antihypertensive effect, whereas amiodarone and dofetilide predominantly influenced systolic pressure-related parameters. Improvements in LV function across all treatment groups—reflected by increased EF and reduced ventricular volumes—suggest partial attenuation of pressure overload-induced dysfunction. However, no major inter-drug differences were observed at the level of global cardiac function, indicating that conventional echocardiographic indices may not fully capture drug-specific myocardial effects. These findings are consistent with previous studies demonstrating that dronedarone attenuates LV hypertrophy and improves coronary vascular remodeling in SHR, in association with enhanced antioxidant capacity, reduced asymmetric dimethylarginine levels, and improved endothelial function [18]. In addition, dronedarone has been shown to modulate hypertrophic signaling pathways, including the nuclear factor of activated T-cells (NFATc4), extracellular signal-regulated kinases 1/2 (ERK1/2), and protein kinase B (AKT), supporting its broader role in structural and molecular cardiac remodeling [19].

Mechanistically, both dronedarone and amiodarone act as multichannel blockers, inhibiting K^+^, Na^+^, and L-type Ca^2+^ currents while exerting antiadrenergic effects. These combined actions contribute to reduced HR and altered autonomic regulation [20,21]. At the cellular level, inhibition of Ca^2+^ and K^+^ currents directly affects excitation–contraction coupling, providing a mechanistic basis for the observed drug-specific differences in myocardial performance [22]. Importantly, despite similar acute electrophysiological properties, dronedarone lacks the pronounced chronic action potential prolongation characteristic of amiodarone, suggesting divergent effects on long-term electrical remodeling [23].

Ex vivo Langendorff experiments further delineated intrinsic myocardial effects independent of systemic influences [24,25]. Under these conditions, dronedarone exerted negative chronotropic and inotropic effects, reducing contractility, HR, and CF, whereas amiodarone demonstrated intermediate effects. In contrast, dofetilide enhanced contractile performance. These findings indicate that intrinsic myocardial responses may diverge substantially despite broadly similar antiarrhythmic classification. Importantly, among the antiarrhythmic agents investigated, dofetilide showed the most consistent results across the functional, structural, and molecular endpoints evaluated in the present model. This observation is particularly noteworthy given its relatively selective IKr-blocking profile compared with the multichannel actions of amiodarone and dronedarone [26,27]. Moreover, the positive inotropic profile of dofetilide may be linked to improved Ca^2+^ handling and reduced oxidative stress, as suggested by experimental models implicating NADPH oxidase-related pathways in excitation–contraction coupling [28,29]. Consistent with these observations, previous studies using isolated heart preparations have shown that dronedarone reduces myocardial contractility while improving tolerance to ischemia-reperfusion injury, indicating a complex pharmacological profile combining negative inotropy with potential cardioprotective effects [24]. In contrast, both amiodarone and dronedarone appear to exert more pronounced functional effects under pathological stress conditions than under basal states [25]. Additionally, their coronary vasodilatory properties in isolated heart models are mediated by NO-dependent and Ca^2+^ channel-related mechanisms, respectively [30]. These differences likely contribute to the observed drug-specific modulation of CF in ex vivo conditions. The apparent discrepancy between in vivo antihypertensive efficacy and ex vivo myocardial performance observed with dronedarone suggests that its systemic hemodynamic benefits are likely mediated predominantly through vascular and neurohumoral mechanisms rather than an intrinsic enhancement of cardiomyocyte contractility [31].

A central finding of the present study is the differential modulation of oxidative stress pathways by chronic antiarrhythmic therapy. HTA is characterized by increased production of reactive oxygen species from enzymatic and mitochondrial sources, which drive redox-sensitive signaling pathways involved in vascular and myocardial remodeling [12]. In this context, both systemic and coronary oxidative stress markers were significantly altered across treatment groups, indicating biologically relevant effects beyond electrophysiological action. Dofetilide and amiodarone were generally associated with a more favorable antioxidant profile, as evidenced by the reduced O_2_^−^ levels and increased GSH content and CAT activity.

In contrast, dronedarone exhibited a more heterogeneous redox response, characterized by reduced NO metabolites but less consistent activation of classical antioxidant defenses. Interestingly, these systemic findings were paralleled by changes observed in CVE during ex vivo perfusion. Dofetilide showed the most consistent reduction in O_2_^−^ production across both systemic and coronary compartments, suggesting a more stable myocardial-specific antioxidant effect compared with the other agents.

At the mitochondrial level, amiodarone has been shown to induce H_2_O_2_ production, cardiolipin peroxidation, and Complex I inhibition, resulting in impaired oxidative phosphorylation and ATP depletion. In contrast, dronedarone induces mitochondrial uncoupling without triggering overt oxidative injury, indicating a dissociation bioenergetic modulation and oxidative damage [32]. Notably, amiodarone also exhibits direct free radical-scavenging activity and protects cardiomyocytes against H_2_O_2_-mediated injury, further supporting its dual role as both a modulator and target of redox processes [33]. Although previous studies in SHR have demonstrated the beneficial effects of dronedarone on oxidative balance and vascular remodeling [18], the less pronounced antioxidant response observed in the present study may reflect differences between systemic vascular and intrinsic myocardial redox regulation, particularly under ex vivo conditions. This may also contribute to its more prominent negative inotropic and chronotropic effects observed in the Langendorff model.

The present histopathological analysis demonstrates pronounced structural remodeling in the myocardium of SHRs, characterized by cardiomyocyte hypertrophy, disorganization of myocardial fibers, interstitial expansion, and vascular wall thickening. These findings are consistent with established features of pressure overload-induced cardiac remodeling, in which chronic hypertension leads to severe structural disarray and hypertrophy of cardiomyocytes, muscle fiber loss, and vacuolar change [34]. Such changes are widely recognized in pressure-overloaded hypertensive hearts as consequences of sustained oxidative stress, where excessive ROS production contributes to cardiomyocyte damage and progression toward heart failure phenotypes [35]. In the present study, antiarrhythmic treatment modified myocardial remodeling to varying degrees. Amiodarone and dronedarone partially attenuated myocardial disorganization and necrotic changes compared with untreated SHRs, although residual structural abnormalities persisted. In contrast, dofetilide-treated animals exhibited the most preserved myocardial architecture, with minimal fibrosis and reduced signs of cardiomyocyte injury, suggesting a more favorable profile with respect to myocardial structural integrity in hypertensive conditions. The observed histopathological differences may be linked to drug-specific effects on oxidative stress and mitochondrial function. This preservation of myocardial structure in the dofetilide group is particularly important, as it suggests a direct link between reduced oxidative stress and the attenuation of pressure-induced remodeling.

Myocardial HSP70 expression further supports the presence of oxidative and cellular stress in hypertensive myocardium. Increased HSP70 expression in untreated SHRs probably reflects the activation of endogenous protective mechanisms in response to chronic stress. This is in agreement with studies demonstrating that HSPs are upregulated in failing and hypertensive hearts as part of a compensatory stress response [36]. Interestingly, reduced HSP70 immunoreactivity in treated groups, particularly in dofetilide-treated animals, may indicate either reduced cellular stress or impaired stress-response capacity depending on the extent of mitochondrial and oxidative modulation induced by therapy. Similar bidirectional regulation of stress proteins has been reported in experimental models of cardiac injury and heart failure progression [36].

SERCA2 expression analysis provided additional insight into functional myocardial integrity. SERCA2 expression was markedly reduced in SHR myocardium, consistent with impaired sarcoplasmic reticulum Ca^2+^ handling and abnormal excitation–contraction coupling [37]. Restoration of SERCA2 expression in treated groups, particularly in the dofetilide group, suggests improved Ca^2+^ handling, which likely contributes to the enhanced contractile performance observed ex vivo. Amiodarone and dronedarone showed intermediate effects, suggesting partial restoration of Ca^2+^cycling, indicating partial recovery and the weaker modulation of Ca^2+^-handling machinery. These findings suggest that beyond ion channel blockade, class III AADs differentially influence intracellular Ca^2+^ regulatory networks, potentially via redox-sensitive posttranslational modification of Ca^2+^-handling proteins.

From a translational perspective, the differential pharmacological profiles identified in this experimental study may have relevance to clinical decision-making in patients with hypertension-related arrhythmias, particularly atrial fibrillation. Amiodarone, dronedarone, and dofetilide are all currently used in clinical practice, yet their selection is primarily guided by electrophysiological considerations, safety profiles, and comorbidities rather than by their effects on myocardial redox balance or Ca^2+^ handling [7]. The findings in our study suggest that oxidative stress modulation and preservation of SERCA2-dependent Ca^2+^ cycling may represent additional pharmacodynamic dimensions that differ substantially among these agents. Whether these differences contribute to clinically meaningful disparities in long-term outcomes, including rates of arrhythmia recurrence, ventricular dysfunction, or adverse remodeling, remains to be established in prospective clinical or translational studies. It should be noted that the SHR model, despite being a widely used and validated model of essential hypertension, differs from human disease in several important respects, including the pace of hypertensive remodeling, neurohumoral activation patterns, and the absence of concomitant pharmacological treatment; these factors may limit the direct clinical applicability of the present findings.

Several limitations of the present study should be acknowledged. The use of the SHR model, which represents a genetic model of essential hypertension, limits the generalizability of the findings to other etiological forms of hypertension, including secondary hypertension or models characterized by concurrent metabolic comorbidities such as obesity or diabetes. Also, the relatively small sample size (n = 6 per group), while consistent with standard practice for Langendorff preparations, may have limited statistical power to detect subtle inter-group differences; some observed numerical trends may therefore reflect biologically meaningful effects that remained below the threshold of statistical significance. Experimental research uses animals that are generally genetically very similar to each other, unlike the human population, as a result of which the translation of the results obtained from experimental studies does not always have the expected effect. The absence of a normotensive control group (e.g., normotensive Wistar Kyoto rats) is one of the limitations. All comparisons are therefore relative to the untreated hypertensive state, and the findings should be interpreted accordingly. Future studies should include a normotensive control group to allow determination of the degree to which each agent restores physiological parameters. The Langendorff model, while valuable for isolating intrinsic myocardial function, does not fully recapitulate in vivo neurohumoral regulation, including sympathoadrenal activation and renin-angiotensin-aldosterone system signaling, which may importantly modulate drug responses in the intact organism. The four-week treatment duration, while sufficient to assess subacute pharmacological effects, may not capture the full extent of long-term remodeling processes associated with chronic antiarrhythmic therapy in hypertensive conditions. Finally, the present study did not include direct electrophysiological measurements, such as surface ECG recordings, QT interval assessment, evaluation of atrioventricular conduction, or proarrhythmia susceptibility testing. Given that the investigated agents are primarily classified and used based on their electrophysiological properties, this represents a significant limitation in the context of translational relevance. The observed structural and redox findings should therefore be interpreted as complementary to, rather than substitutes for, electrophysiological characterization. Future studies incorporating in vivo electrophysiological assessments are warranted to provide a more comprehensive pharmacological profile of these agents in the hypertensive heart.

Taken together, these findings support the concept that class III AADs exert pleiotropic effects on myocardial redox homeostasis, which may in turn modulate both electrical stability and mechanical performance. Such redox-dependent mechanisms are likely to be particularly relevant under conditions of chronic cardiac stress, where oxidative imbalance contributes to arrhythmogenesis and progressive ventricular dysfunction.

## 4. Materials and Methods

### 4.1. Ethical Approval

This study was carried out in the Center of Excellence for the Study of Redox Balance in Cardiovascular and Metabolic Disorders, Faculty of Medical Sciences, University of Kragujevac, Kragujevac, Serbia. All experimental procedures were performed in accordance with the principles of the European Directive for the Welfare of Laboratory Animals, No: 2010/63/EU, and the principles of Good Laboratory Practice. Animals were cared for in accordance with the Guide for the Care and Use of Laboratory Animals (National Research Council 1996) and the principles of Good Laboratory Practice (GLP). The protocol was approved by the Ethics Committee for Experimental Animal Wellbeing (No: 01-977/2. date 30.01.2019) of the Faculty of Medical Sciences, University of Kragujevac.

### 4.2. Animals and Experimental Design

**Figure 9:**
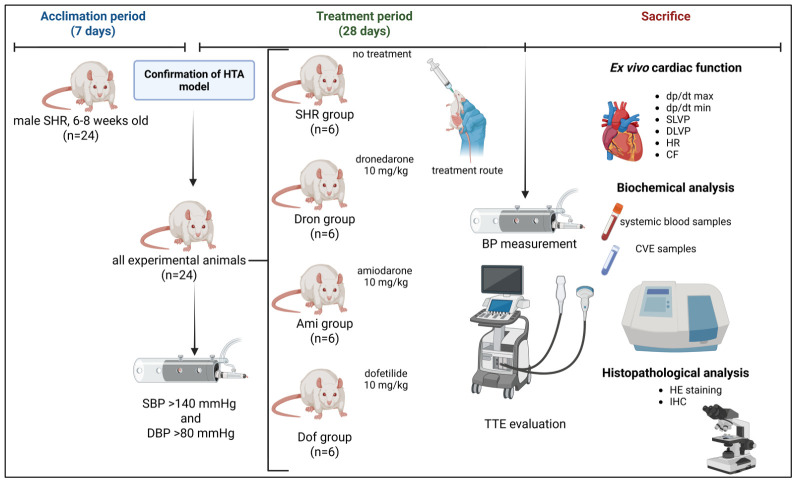
Experimental study design. Abbreviations: n—number of animals; SHR—spontaneously hypertensive rat; Dron—dronedarone; Ami—amiodarone; Dof—dofetilide; TTE—transthoracic echocardiography; BP—blood pressure; HE—hematoxylin and eosin; ICH—immunohistochemistry. Created in BioRender: Srejovic, I. (2026) https://BioRender.com/byuszc8, accessed on 20 June 2026.

A total of 24 male SHRs, aged 6–8 weeks and with a body weight (BW) of 180 ± 20 g, were obtained from the Animal House of the Military Medical Academy (Belgrade, Serbia). Animals were housed in polyethylene cages (six per cage) under standardized, controlled environmental conditions (room temperature 21 ± 2 °C, humidity 55 ± 5%, and 12-h dark–light cycle), with ad libitum access to standard chaw (20% protein rat food) and water. The experimental study design is presented in Figure 9.

Following a one-week acclimation period, HTA (defined as systolic blood pressure (SBP) > 140 mmHg and diastolic blood pressure (DBP) > 80 mmHg) was confirmed in all animals before inclusion in the experimental protocol using the non-invasive tail-cuff method (Rat Tail Cuff Method Blood Pressure Systems (MRBP-R), IITC Life Science Inc., Los Angeles, CA, USA). Afterward, all animals were randomly assigned to one of the following groups according to the applied treatment protocol: SHR group (untreated group receiving saline at equivalent dose daily), Dron group (receiving dronedarone at a dose of 10 mg/kg daily), Ami group (receiving amiodarone at a dose 10 mg/kg daily), and Dof group (receiving dofetilide at a dose 10 mg/kg daily). All treatments were administered by oral gavage and treatment duration was 4 weeks. Dosing regimens were in accordance with previous studies [38,39,40].

### 4.3. Assessment of In Vivo Heart Function

As described previously, SBP and DBP were measured using a non-invasive tail-cuff method at two time points: (1) before the inclusion of animals in the study (to confirm HTA) and (2) at the end of the experimental protocol, after 4 weeks of drug administration, one day before sacrifice.

Transthoracic echocardiography (TTE) was performed just before sacrifice using a Hewlett Packard Sonos 5500 ultrasound system (Andover, MA, USA) equipped with a 15.0 MHz phased-array transducer. Rats were anesthetized by inhalation anesthesia (RWD Life Science Co., Ltd., Shenzhen, China) with isoflurane, placed in the appropriate supine position, and their chests were shaved to perform TTE examination in M-mode and two-dimensional (2D) mode. All measurements were averaged from three consecutive cardiac cycles during M-mode tracing. The following parameters of LV structure and function were assessed: intraventricular septal wall thickness at end-diastole (IVSd) and end-systole (IVSs), LV internal diameter at end-diastole (LVIDd) and end-systole (LVIDs), and LV posterior wall thickness at end-diastole (LVPWd) and end-systole (LVPWs). LV end-diastolic volume (LVEDV), LV end-systolic volume (LVESV), fractional shortening (FS), and ejection fraction (EF) were calculated using a previously established formula [41].

### 4.4. Assessment of Ex Vivo Heart Function

All animals were sacrificed by decapitation using a guillotine for small laboratory animals. Immediately after decapitation, systemic blood was collected for further biochemical analyses, and the thorax was opened and prompt cardiac excision was performed. After excision, the hearts of all animals were immersed in ice-cold saline, aiming to reduce the oxygen demand of myocardium and ischemic heart damage. Upon cannulation of the aortas, retrograde perfusion was established, enabling nutrition of myocardium. The time from animal sacrifice to the establishment of retrograde perfusion did not exceed 30 s.

The hearts were perfused with complex Krebs–Henseleit buffer, the composition of which was as follows (mmol/L): NaCl—118; KCl—4.7; CaCl_2_·2H_2_O—2.5; MgSO_4_·7H_2_O—1.7; NaHCO_3_—25; KH_2_PO_4_—1.2; glucose—11; pyruvate—2, equilibrated with 95% O_2_ plus 5% CO_2_, and warmed to 37 °C (pH 7.4).

Immediately after the establishment of spontaneous cardiac activity, an incision was made in the left atrium, the mitral valves were interrupted, and a sensor for continuous monitoring of cardiodynamic parameters was placed in the LV. Coronary perfusion pressure (CPP) was adjusted to 70 cmH_2_O during the stabilization period, lasting for 25 min. The cardiodynamic parameters measured during the experiment were the following: maximum rate of pressure development in the LV (dp/dt max), minimum rate of pressure development in the LV (dp/dt min), systolic LV pressure (SLVP), diastolic LV pressure (DLVP), and heart rate (HR). Coronary flow (CF) was measured flowmetrically. After establishment of stable cardiac activity (measured CF was the same in three repeated measurements) at a CPP of 70 cmH_2_O, CPP decreased to 60 cmH_2_O, then gradually increased to 120 cmH_2_O (80 cmH_2_O, 100 cmH_2_O and 120 cmH_2_O), and again immediately decreased to 40 cmH_2_O. At every CPP, heart function was recorded for minimum 5 min and values of the cardiodynamic parameters were obtained, indicating the ability of the myocardium and coronary circulation to adapt to CPP changes [42]. Coronary venous effluent (CVE) was collected at every CPP for further biochemical analysis.

### 4.5. Oxidative Stress Parameters in Systemic Circulation and Coronary Venous Effluent

Oxidative stress parameters were assessed by a Shimadzu UV 1800 spectrophotometer (Kyoto, Japan) in systemic blood samples (plasma and erythrocyte lysate) and CVE.

As previously described, venous blood samples were collected immediately after sacrifice. Plasma and erythrocytes were separated by centrifugation. Plasma samples and samples of erythrocyte lysate were prepared and stored at −80 °C until analysis, as previously described [43]. The following biomarkers were analyzed in plasma: index of lipid peroxidation (expressed as thiobarbituric acid reactive substances (TBARSs)), nitric oxide (NO) in the form of nitrites (NO_2_^−^), superoxide anion radical (O_2_^−^), and hydrogen peroxide (H_2_O_2_). In addition, the activities of superoxide dismutase (SOD), catalase (CAT), and reduced glutathione (GSH) were determined in erythrocyte lysate.

During the ex vivo assessment of heart function, samples of CVE were collected at each CPP and stored at −80 °C until analysis, as previously described [44]. The following OS markers were determined: TBARS, NO_2_^−^, O_2_^−^, and H_2_O_2_.

The index of lipid peroxidation, measured as TBARS, was determined for the quantification of lipid peroxidation in plasma and CVE. Thiobarbituric acid (1%) dissolved in 0.05 NaOH was incubated with plasma or CVE samples at 100 °C for 15 min. The absorbance was measured at a wavelength of λ = 530 nm. Distilled water was used as a blank for plasma, and Krebs–Henseleit solution was used as a blank for CVE [44,45]. Blanks were the same in all oxidative stress biomarkers measurements in plasma and CVE.

NO_2_^−^ determination was used for the assessment of NO in plasma and CVE samples. Griess’s method was applied for the determination of NO_2_^−^, using freshly prepared Griess’s reagent. The sample was precipitated with 30% sulfosalicylic acid, and then the resulting mixture was centrifuged. Equal volumes of supernatant and Griess’s reagent were mixed, followed by incubation for 10 min in the dark. The absorbance was measured at a wavelength of λ = 543 nm [44,45].

O_2_^−^ determination was based on the reaction of nitro blue tetrazolium (NBT) in tris (hydroxymethyl) aminomethane (TRIS)-buffer with O_2_^−^ plasma or CVE samples. During the above reaction, nitro formazan blue is formed. The absorbance was measured at a wavelength of λ = 530 nm [44,45].

Spectrophotometric quantification of H_2_O_2_ was based on the oxidation of phenol red by H_2_O_2_ in a reaction catalyzed by horseradish peroxidase (HRPO). The sample (plasma or CVE) was precipitated with freshly prepared phenol red solution, and then the ex-tempore prepared solution of HRPO was added. The absorbance was determined at a wavelength of λ = 610 nm [44,45].

The activity of SOD, CAT, and the amount of GSH were determined in erythrocyte lysate. SOD activity determination was based on the epinephrine method, and detection was performed at λ = 470 nm. CAT activity was determined according to Beutler’s method. Erythrocyte lysate samples were diluted with distilled water and treated with chloroform-ethanol to remove hemoglobin, and then the sample was mixed with CAT buffer and H_2_O_2_. The absorbance was determined at a wavelength of λ = 360 nm, while distilled water was used as the blank. Determination of GSH was based on Beutler’s method on GSH oxidation by 5,5-dithiobis-6,2-nitrobenzoic acid. The absorbance was determined at a wavelength of λ = 420 nm [42].

### 4.6. Histopathological Analysis

After completion of the assessment of ex vivo cardiac function, all hearts were fixed in 4% buffered formalin, embedded in paraffin blocks, sectioned into 5 µm slices, and stained using HE according to standard histological protocols. Micromorphological analysis of myocardial tissue was performed using light microscopy at ×200 original magnification. The degree of morphological changes in myocardial tissue was assessed using a semi-quantitative scale on HE-stained sections, performed by a pathologist blinded to group allocation (blinded scoring). For each animal, 10 visual fields were analyzed, randomly selected from 6 transverse myocardial sections, at a magnification of 200×. The following morphological parameters were evaluated: myofibrillar disarray (disorganization and waviness of myofibrils), interstitial edema/widening of the interstitial space, vacuolization of cardiomyocyte cytoplasm, inflammatory cell infiltration, and changes in nuclear size and shape. Each parameter was scored on a scale from 0 to 3: 0—no changes (normal architecture); 1—mild changes (focal, <25% of the visual field); 2—moderate changes (25–50% of the visual field); 3—severe changes (>50% of the visual field). The total score for each visual field represented the sum of the scores for all parameters, and the mean value per group was expressed as a percentage relative to the SHR group, which was defined as 100%.

For immunohistochemical (IHC) analysis, paraffin-embedded myocardial sections were deparaffinized in xylene and rehydrated through graded ethanol solutions. Antigen retrieval was performed using citrate buffer (pH 6.0) under heat-induced conditions. Endogenous peroxidase activity was blocked with 3% H_2_O_2_, followed by incubation with blocking solution to reduce nonspecific antibody binding.

To assess oxidative stress-related cellular response, sections were incubated with mouse monoclonal anti-HSP70 antibody (HSP70 (3A3), sc-32239, Santa Cruz Biotechnology, Dallas, TX, USA) at an appropriate dilution according to the manufacturer’s instructions. HSP70 immunoreactivity was visualized predominantly as cytoplasmic staining in cardiomyocytes, as previously described [46]. To evaluate proteins involved in myocardial Ca^2+^ handling and contractile function, sections were incubated with mouse monoclonal anti-SERCA2 antibody (SERCA2 (IID8), sc-53010, Santa Cruz Biotechnology, Dallas, TX, USA). SERCA2 immunostaining was analyzed as cytoplasmic positivity in myocardial cells, as previously described [47].

After incubation with primary antibodies, sections were treated with appropriate secondary antibodies and visualized using 3,3′-diaminobenzidine (DAB) chromogen. Finally, sections were counterstained with hematoxylin, dehydrated, and mounted for microscopic evaluation. Quantification of HSP70 and SERCA2 immunoreactivity was performed by two independent pathologists blinded to group allocation. Digital images were captured using light microscopy at ×200 magnification and analyzed using ImageJ software 1.54g (National Institutes of Health, Bethesda, MD, USA). Color deconvolution with the H-DAB vector was applied to separate the DAB-positive (brown) channel from the hematoxylin counterstain. The percentage of DAB-positive myocardial area relative to the total analyzed tissue area was determined for each field at ×200 magnification. For each animal, 10 visual fields were analyzed, randomly selected from 6 transverse myocardial sections. The primary observer performed the analysis, and all results were independently verified by a second blinded observer; discrepancies were resolved by consensus. The mean value per group was expressed as a percentage relative to the SHR group, which was defined as 100%. Representative photomicrographs were obtained using light microscopy at ×200 original magnification.

### 4.7. Statistical Analysis

Prior to hypothesis testing, all datasets were evaluated for distribution normality using the Shapiro–Wilk test. Depending on the outcome, appropriate parametric or non-parametric procedures were applied. For normally distributed variables, group differences were assessed using analysis of variance (ANOVA), followed by Tukey’s post hoc test for multiple comparisons. For variables that did not satisfy normality assumptions, group differences were assessed using the Kruskal–Wallis non-parametric test, followed by Dunn’s post hoc test for pairwise comparisons. Data are presented as the mean ± standard deviation (SD), and statistical significance was accepted at *p* < 0.05. Statistical analyses were performed using GraphPad Prism 10 for MacOS.

The present study employed 6 animals per experimental group, which is consistent with the standard practice in isolated heart perfusion studies that involve technically demanding procedures [48,49]. However, it should be acknowledged that this sample size may have limited the statistical power to detect subtle differences between treatment groups, and some observed trends may not have reached statistical significance due to inadequate power.

## 5. Conclusions

In conclusion, the present study demonstrates that class III AADs exert distinct and multi-faceted effects on myocardial performance, structural integrity, and redox homeostasis in hypertensive conditions. Importantly, despite sharing class III antiarrhythmic classification, these agents exhibit markedly different cardiometabolic profiles that extend beyond their electrophysiological actions. Dofetilide and amiodarone preferentially enhance antioxidant capacity, preserve myocardial structure, and improve Ca^2+^ handling, whereas dronedarone exerts a more pronounced antihypertensive effect accompanied by reduced intrinsic contractile performance in ex vivo conditions. Among all tested agents, dofetilide showed the most consistent results across the functional, structural, and molecular endpoints evaluated in this experimental model. These findings should be interpreted in the context of the study’s scope, as no direct electrophysiological assessments were performed. These functional differences are supported by histological and immunohistochemical findings, where dofetilide demonstrated the greatest preservation of myocardial architecture and SERCA2 expression, while untreated hypertensive hearts exhibited marked structural remodeling and elevated HSP70 expression, reflecting increased cellular stress. Amiodarone and dronedarone partially attenuated these alterations, indicating intermediate cardioprotective effects. Taken together, these results suggest that improvement of redox balance and preservation of Ca^2+^-handling machinery may represent central determinants of myocardial protection in hypertension, beyond simple ion channel blockade. These pleiotropic actions, particularly those related to oxidative stress modulation, may play a critical role in shaping both mechanical performance and arrhythmogenic susceptibility under conditions of chronic cardiac stress. Future studies should aim to integrate molecular, electrophysiological, metabolic, and mitochondrial analyses in order to further elucidate the interplay between ion channel modulation and redox-sensitive signaling pathways. Translational investigations in clinically relevant models are warranted to determine whether these drug-specific effects contribute to differences in long-term therapeutic efficacy, safety, and potential disease-modifying properties.

## Data Availability

The original contributions presented in this study are included in the article. Further inquiries can be directed to the corresponding authors.

## Abbreviations

The following abbreviations are used in this manuscript:

AADs	Antiarrhythmic drugs
ANOVA	Analysis of variance
AF	Atrial fibrillation
AKT	Protein kinase B
Ami	Amiodarone
BP	Blood pressure
CAT	Catalase
CVE	Coronary venous effluent
DAB	3,3′-diaminobenzidine
DBP	Diastolic blood pressure
Dof	Dofetilide
Dp/dt max	Maximum rate of pressure development in the left ventricle
Dp/dt min	Minimum rate of pressure development in the left ventricle
DLVP	Diastolic left ventricular pressure
Dron	Dronedarone
EF	Ejection fraction
ERK	Extracellular signal-regulated kinase ½
FS	Fractional shortening
GLP	Good Laboratory Practice
GSH	Reduced glutathione
H_2_O_2_	Hydrogen peroxide
HE	Hematoxylin and eosin
HF	Heart Failure
HFrEF	Heart failure with preserved ejection fraction
HR	Heart rate
HRPO	Hore radish peroxidase
HSP70	Heat shock protein 70
HTA	Arterial hypertension
ICH	Immunohistochemistry
IHD	Ischemic heart disease
IVSd	Intraventricular septal thickness at end-diastole
IVSs	Intraventricular septal thickness at end-diastole
NBT	Nitro blue tetrazolium
NFATc4	Nuclear factor of activated T-cells
NO	Nitric oxide
NO_2_^−^	Nitric oxide levels expressed as nitrites
NOS	Nitric oxide synthase
O_2_^−^	Superoxide anion radical
LVEDV	Left ventricular end-diastolic volume
LVESV	Left ventricular end-systolic volume
LVIDd	Left ventricular internal diameter at end-diastole
LVIDs	Left ventricular internal diameter at end-systole
LVPWd	Left ventricular posterior wall thickness at end-diastole
LVPWs	Left ventricular posterior wall thickness at end-systole
LVH	Left ventricular hypertrophy
RAAS	Renin-angiotensin-aldosterone system
ROS	Reactive oxygen species
RNS	Reactive nitrogen species
RyRs	Ryanodine receptors
SBP	Systolic blood pressure
SD	Standard deviation
SERCA	Sarcoplasmic reticulum Ca^2+^ATPase
SHRs	Spontaneously hypertensive rats
SLVP	Systolic left ventricular pressure
SOD	Superoxide dismutase
TBARS	Index of lipid peroxidation
TRIS	Trys (hydroxymethyl) amino methane
TTE	Transthoracic echocardiography
